# Hate, crime and epistemic vulnerability: on sense-making and feelings of (un)safety among Danish Muslims

**DOI:** 10.3389/fsoc.2024.1347803

**Published:** 2024-06-18

**Authors:** Anne-Mai Flyvholm, Birgitte Schepelern Johansen

**Affiliations:** Department of Cross-Cultural and Regional Studies, University of Copenhagen, Copenhagen, Denmark

**Keywords:** hate crime, epistemic practices, sense of safety, sense-making, hermeneutical practices, testimonial practices

## Abstract

This article investigates feelings of (un)safety emerging from knowing and sharing knowledge about hate crime and hate incidents. Drawing on fieldwork and interviews with young Muslims living in the greater Copenhagen area, the article explores the way the interlocutors seek to make sense of their experiences through available epistemic categories, and how this sense-making is shaped by reactions from the surrounding society, e.g., whether it is questioned, supported, ignored etc. Combining criminological and psychological research on direct and indirect harms of hate crime with insights from philosophy on epistemic encounters and their ethical implications the article provides a framework for investigating safety in epistemic interactions. Based on this framework, the article show the often hard work that people perform in order to balance epistemic needs (e.g. the need for knowledge and for recognition) with epistemic risks (e.g. the risk of testimonial rejection, of damaged epistemic confidence, or loss of credibility).

## 1 Introduction

To know and to share knowledge can indeed be risky endeavors. This is not least true, when the object of knowledge is hate crime and hate incidents. Research into the effects of hate crime shows, among other things, that learning about hate-motivated offenses against people, with whom you identify or with whom you share identifiable traits, can foster experiences of unsafety, anxiety, and vulnerability as well as anger and indignation (Noelle, 2002; Perry and Alvi, 2011; Paterson et al., 2019b). Obviously, such reactions to hate crime do not occur in a social and political void. They emerge within specific situations, places and interactions, in which people try to make sense of experiences with and stories about hate crime and hate incidents, and in which these attempts to make sense are met, acknowledged, contested, ignored or even rejected by others. This socially embedded epistemic work of making sense entails risks of its own. Hate crime and hate incidents usually occur along the lines of prevailing social hierarchies, based on for example gender, sexual orientation, religion or race (Perry, 2001; Chakraborti and Garland, 2012). For this reason, they are likely to tap into already existing structural vulnerabilities, which are likely to affect epistemic resources such as credibility and the ability to speak with effect in public contexts (Fricker, 2007). Being vulnerable to hate crime and hate incidents is thus likely to go hand in hand with various forms of epistemic vulnerability. At the same time, if not properly recognized hate crime and hate incidents may affect those targeted, damaging their confidence in public authorities, mainly the police (Christmann and Wong, 2010; Chakraborti and Hardy, 2015). For this reason, the conditions for knowing – that is: the conditions for making one’s experiences of and reactions to hate crime and hate incidents intelligible to oneself as well as to relevant others – becomes particularly acute.

In this article, we wish to engage this epistemic dimension of senses of safety and explore feelings of (un)safety emerging from knowing and sharing knowledge about hate crime or hate incidents, whether from personal experience or other sources. Drawing on fieldwork and interviews with young Muslims living in the greater Copenhagen area, we wish to explore the way our interlocutors seek to make sense of their experiences through available epistemic categories, and how this sense-making is shaped by reactions from the surrounding society, e.g., whether it is questioned, supported, ignored etc.

## 2 Hate crime and senses of (un)safety

On a minimal understanding, safety can be seen as a state of being secure from harm and threat of harm (Veale et al., 2023). Hate or bias-related crime – that is, criminal acts that are motivated by prejudice such as racism, antisemitism, homophobia or transphobia – is likely to perpetuate harm across several domains in a person’s life. Being targeted by hate crime potentially implies threats not just to a person’s physical safety, but also one’s social and moral standing, as well as psychological and emotional well-being. Research into the consequences of hate and bias crime has established a number of damaging repercussions of hate crime victimization. In a seminal study among LGBT persons living in the Sacramento area, Gregory Herek et al. (1999) showed that victims of hate crime suffer heightened levels of depression and anxiety, diminished feelings of safety and self-mastery, and an increased tendency to attribute personal set-backs to societal prejudices. Specifically, granted that hate crimes mainly target persons who carry minoritized identity markers, victimization seems to create a difficult terrain for navigating one’s safety:

*Hate-crime victimization may upset the balance of, on the one hand, the need to maintain an adaptive illusion of personal invulnerability and relative safety from persecution based on one's minority status and, on the other hand, the need to realistically appraise situations that might pose a danger to oneself* (Herek et al., 1999, p. 950).

Other studies have shown similar psychological sequelae, as well as increased feelings of vulnerability to repeat victimization, heightened attentiveness when walking in public spaces, and moderation of appearance so as to become less visible as a minority as ways of coping with feelings of unsafety (McDevitt et al., 2001, also Perry and Alvi, 2011). Many of these consequences are well-known from criminal victimization more generally. However, there is a growing amount of research that indicate that hate crime is more harmful to victims’ senses of safety than comparable non-bias crimes (Iganski, 2001; McDevitt et al., 2001; Iganski and Lagou, 2015 for a comprehensive overview of literature on the various harms of hate crime, see Walters, 2022). There are different explanations for this, and they point toward the bias component of hate crime, which creates particular forms of social and moral harms. Firstly, hate crime targets the victim’s identity, sending a message that ‘people like you’ are inferior, unwanted, disgusting etc. (Perry, 2001). This may cause feelings of anger, anxiety and shame (Paterson et al., 2019b) as well as a heightened sense of social vulnerability beyond what is normally found in crime victims (Lawrence, 2007). Further, as Linda Garnets et al. (1990) have argued, by targeting the victim’s identity, hate crime links something which ought to be a source of stability and confidence to experiences of threat and inferiority. This potentially harms victims’ self-esteem and undermines an important source of strength in processes of coping. Also, an important aspect of hate crime is the potential interchangeability of the victim: that one has been targeted due to a collective identity trait, which (i) positions one as a member of an abstract category of people, and which (ii) is likely to be outside one’s control (McDevitt et al., 2001). This entails a dispersion of threat also to others who share the same identity: that it might as well have been them. Several scholars have argued that hate and bias-related crime, qua ‘message crime’, has intimidating effects beyond the immediate victim (Weinstein, 1992; Iganski, 2001). There is by now a growing body of empirical evidence that supports the hypothesis that the very fact of learning about hate crime committed against people, with whom you identify, may produce some of the same types of reactions as direct victimization (Noelle, 2002; Perry and Alvi, 2011; Paterson et al., 2019a,b; Walters et al., 2020).

To know and to share knowledge about hate crime seems, then, to constitute a potential threat to feelings of safety in itself: for direct victims as well as people who share identity with direct victims. At the same time, knowing and sharing knowledge about hate crime is essential to the ability to give testimony and hence to processes of recognition (legal as well as social), which again may strengthen victims’ ability to go on. It is therefore important to investigate in more details the stakes, risks and gains of epistemic practices surrounding hate crime, in order to gain a more nuanced understanding of the way knowledge is implicated in shaping senses of safety.

## 3 Epistemic needs

Feeling safe has epistemic preconditions: what one knows about one’s surroundings, one’s position, and one’s capacities is likely to shape feelings of safety in a given situation. For example, knowing that people ‘like me’ can become targets of hate crime, may affect how safe one feels in certain places and in the company of certain people. This basic insight is part of the overall framework for our investigation, and we will address this aspect on several occasions during the analysis. However, we also want to hone in on something more specific, namely the senses of safety that relates more narrowly to epistemic practices. This kind of safety pertains to the person *as a knower* and the needs one might have in that capacity. Taking inspiration from philosopher Miranda Fricker’s work on epistemic injustice (2007), we want to focus on the needs and harms associated with two different but related types of epistemic practices, namely (i) to convey knowledge to others (testimonial practices) and (ii) to make sense of one’s experiences by rendering them intelligible through shared epistemic resources (hermeneutic practices). As will be evident below, these two practices are often intertwined, but we still conceptualize them separately, because they imply slightly different needs and harm.

So what kind of needs attaches to testimonial practices? Obviously, there are functional needs: the ability to share knowledge allows human beings to benefit from observations and experiences made by others, which greatly enhance human capacities. To that end, creating structures for sharing and assessing knowledge is indispensable for the development of functioning human societies. However, there are important epistemic needs beyond efficiency and capacity. To the extent that knowledge is also a social currency that confirms our living in a shared reality, being adequately recognized in one’s capacity as a knower is to be recognized as a person with a certain standing (also Congdon, 2018). When the topic at hand is experiences of hate crime and hate incidents, such recognition may obviously pertain to the ability to give testimony about what has happened to relevant others, e.g., the police, family, and colleagues. Adequately recognizing others as knowers in testimonial encounters is, then, an important need for, at least, two reasons. It is important for the quality of our shared knowledge: if people are not believed when telling something truthful, important information is lost to the deficit of an entire community or society (Medina, 2013). Further, it is important for the recognition of the other as a person with a status to know and be heard. To feel safe as a knower in regards to testimonial practices could then, at least as a minimum, require that a person feels certain that attempts to convey what one knows, are taken seriously by other people. To take seriously does not amount to simple agreement, and it does not imply that the knowledge is immune to criticism or correction. People may be unclear, imprecise or mistaken in what they convey. The relevant safety here pertains to the *status* of the person speaking, namely as someone who is owed a fair judgment as to the knowledge conveyed. What we mean by feeling safe in linguistic exchanges, then, ultimately hinges on what Eamonn Callan calls dignity safety, which implies “to be free of any reasonable anxiety that others will treat one as having an inferior social rank to theirs” (Callan, 2016, p. 65).^1^

Human beings also have a need to know and understand in the first place. As anthropologist Fredrik Barth has put it, knowledge is more broadly involved in our ability to relate to and act upon the world around us:

*“We all live lives full of raw and unexpected events, and we can grasp them only if we can interpret them – cast them in terms of our knowledge, or best, anticipate them by means of our knowledge, so we can focus on them and meet them to some extent prepared and with appropriate measures”* (Barth, 2002, p. 1).

To know is a fundamental prerequisite for our ability to make sense of the world and thereby know ‘how to go on.’ In some situations, epistemic resources are already available, but in other situations, new categories and new social understandings have to be crafted. When dealing with hate crime and hate incidents, the very act of asserting an incident as a wrong of this particular kind requires epistemic resources that allows a person to recognize the relevant incident according to established moral and perhaps even legal standards. It also requires confidence in one’s capacity to pass epistemic judgments on one’s experiences, and sometimes it requires the establishment of new interpretive resources to make phenomena hitherto marginalized from socially accepted knowledge intelligible (Fricker, 2007, chap. 7).

To craft certainty that something is real is ultimately a social endeavor that requires a shared language and recognition by relevant others. So one should not think of hermeneutic practices as internal, while testimonial practices are expressive or external. Rather they are both social, and they may take both internal and external forms. Further, hermeneutic practices will frequently take the form of testimonial practices, yet of a more tentative kind: we search for the right words, try out new terms, new categories, seeking resonance and validation for our suggestions by telling others what (we think) might be the case. Still, we may differentiate analytically between them and maintain that the need implied in hermeneutic practices is not so much about being believed as it is about becoming intelligible. So to feel safe as a knower in relation to hermeneutic practices would imply – at least as a minimum – to feel relatively secure in one’s ability to render one’s experiences communicatively intelligible through shared hermeneutical resources, and, if such resources are not available, that there is a collective interest in helping to create them.

## 4 Epistemic harms

Epistemic needs can be harmed or threatened in different ways. Quite a lot has been written on the potential harms that may emerge in linguistic exchanges hampered by identity prejudice and structural inequalities (Fricker, 2007; Walker, 2007; Dotson, 2011; Medina, 2013). Obviously, identity prejudices are themselves epistemically harmful for the person harboring them, however here we focus on the harms or threats of harm for the person who is targeted by the prejudice. For example, identity prejudices such as racism may influence how much credibility people are assigned in linguistic exchanges, what Fricker calls an unfair ‘credibility deficit’ (Fricker, 2007, p. 17). Such unfair credibility assessments may result in different practices, which all have the consequence of damaging the transmission of knowledge. Of particular relevance for the present investigation is Kristie Dotson’s elaboration on practices of silencing (2011). At the heart of Dotson’s account is the idea of speaker dependency – an idea she borrows from Hornsby and Langton (1994, p. 238). Speaker dependency implies that a successful attempt to convey knowledge ultimately depends upon the hearer’s willingness and capacity to listen appropriately. Thus, testimonial exchanges are always marked by a certain vulnerability on behalf of the speaker, who inevitably takes a risk when attempting to convey knowledge. Silencing occurs when hearers refuse to meet this vulnerability and reciprocate a linguistic exchange appropriately, due to pernicious ignorance. Such refusal could be caused by negative stereotypical images, as are often at stake in interactions characterized by identity prejudice. Dotson here gives the example of the stereotyped black woman as either mammie, mother, matriarch or whore, which all work to undermine her as a rational, accountable knower (Dotson, 2011, p. 24). In the context of the present investigation, images of the minoritized person as overly sensitive to racism would be a case in point. But the failure to reciprocate appropriately could also emerge from systematic ignorance of the social reality of minoritized persons, e.g., experiences of racism or discrimination, that work to discredit the person giving testimony.

When speakers are systematically denied appropriate recognition as knowers in linguistic exchanges, it may cause speakers to alter their linguistic practices in order to avoid being rejected, questioned or offended. This could for example imply avoiding interaction with certain people or avoiding or amending one’s speech on certain topics with certain people, what Dotson calls testimonial smothering (Dotson, 2011, p. 244). On top of the personal costs to feelings of safety in linguistic exchanges, testimonial smothering may create rifts or gaps in the shared knowledge about the worlds in which we live together.

The need for adequate epistemic resources that allow us to cast our experiences in terms of shared, recognized and recognizable knowledge can also be harmed or threatened in various ways. A society’s epistemic resources are of course shaped in numerous contexts and through various institutional arrangements, crucial among these, schools and various institutions for higher education and research, but also through public venues for communication, such as mass media and social media (Barth, 2002). One important way in which access to shared epistemic resources can be threatened is if knowledge about certain topics, certain people, certain historical events etc. is systematically excluded from such institutions or distorted in the way they are represented. As Fricker points out, a society’s epistemic resources are shaped by power relations, so they are often better suited to grasp the interests and experiences of those in power (Fricker, 2007, p. 147). Epistemic resources are also continuously developed through daily interactions and attempts to make sense of our experiences. Harm can be done to this kind of hermeneutic work, when a person is routinely doubted or dismissed in their attempts to put experiences, intuitions or sensations into words. Ultimately, doubt and dismissal can harm a person’s epistemic confidence, or what Fricker calls “intellectual courage” (Fricker, 2007, p. 49), which again may cause self-doubt and hamper the possibilities for sharing experiences and crafting common responses to them. In the following analysis, we will draw on this conceptual vocabulary in order to investigate experiences with linguistic encounters about hate crime and hate incidents among our interlocutors and explore the senses of (un)safety and risk that emerge from these encounters. As part of this, we explore the hermeneutic work people perform in order to make sense of their own and others’ experiences with potential hate crimes: how they search for an appropriate language and how interactions with others (witnesses, friends, family, colleagues, but also the police) shape their feelings of safety regarding their abilities to assert what is the case.

## 5 Materials and methods

The analysis in this article draws on data gathered through 13 in-depth interviews with Muslims from the greater Copenhagen area, Denmark, and fieldwork at a Copenhagen mosque. The study is part of a larger research project into the wider social impacts of hate crime, which combines fieldwork and interviews with Muslims and Jews living in Copenhagen with a study of media reporting and political discourses on hate crime during the last 20 years. The ‘wider’ impacts are understood in two different ways: Firstly, the project probes the consequences of hearing of or reading about hate crime committed against people with whom our interlocutors share identity traits. The interlocutors for the study were not recruited based on whether or not they had direct experiences with hate crime, since the initial interest of the project was in the indirect impacts of hate perpetration. However, throughout most interviews, though the interviewer only asked about indirect experiences of hate incidents, direct experiences with racist or islamophobic victimization were brought up alongside indirect experiences. Secondly, the project also probes the possible consequences of these experiences of immediate victimization, and how they shape subsequent reactions to hearing about hate crime. The data gathered for this study shows that narratives of direct as well as indirect experiences of hate incidents are woven together to form a coherent perception of hate crime. This is by no means to say that experiencing a hate crime and hearing about it, is the same thing, as also shown in previous research by Paterson et al. (2019b). However, in the subsequent analysis, we aim to respect the way the two types of experience, however different, for our interlocutors are intertwined and colors one another.

Participants for interviews were reached through institutions and organizations, which primarily address Danish Muslims, including a mosque (5), a Muslim elementary school (4), and a Danish Muslim Rights organization (1). In addition, respondent driven sampling was successful in a few cases (3). One interview was conducted with a young woman, who posts about experiences of Muslims in Denmark on social media. She was included in the project based on references from other interlocutors to her social media profile. In the analysis below, all participants are given pseudonyms. The recruitment process resulted in 13 in-depth interviews lasting between 1 and 3 h. Participants varied in gender, age, occupation and educational background in the following ways: Interlocutors were between 21 and 42 years old at the time of the interviews. This rather young sample of respondents has likely resulted from the primary places of recruitment: The Danish department of the mosque, which attracts mostly young Muslims as well as parents at Muslim elementary schools, who are likely in their 30s and 40s with elementary school aged children. Attempts were made to include a wider age range through respondent driven sampling, which, however did not prove successful. The educational background and occupation of the interlocutors ranged from professional bachelors (such as social worker) to masters degrees, and interlocutors were either without job (1), students or recent graduates (6), in occupation (6). In terms of gender, the sample is skewed with 11 women and 2 men. The gender bias is partly due to the initial fieldwork in the mosque, where Flyvholm participated in the women’s section of the mosque. However, subsequent contact to institutions and organizations was not limited in this way. The gender imbalance may also have to do with the researcher conducting the fieldwork being a woman herself. Finally, quite a few interlocutors talked about Muslim women being more exposed or vulnerable to hate perpetration because of their heightened visibility as Muslims when wearing the veil. Though it is not possible to confirm based on the data gathered for this study, it is possible that such a heightened sense of vulnerability among Muslim women could have been a motivating factor for participating in a study such as this. In the research literature on Muslims in Europe, there is a call for caution not to make “Muslims all about Islam” (Jeldtoft and Nielsen, 2011; Brekke et al., 2019). There is a high diversity in the ways in which individuals identifying as Muslims practice and identify as Muslims and whether and how closely they are affiliated with religious organizations, and this should be reflected in the research. The present research project has attempted to account for this diversity by recruiting participants through both religious organizations (such as a mosque), non-religious organizations that cater primarily to Muslims in Denmark (such as schools that are not Islamic but are used by Muslim families who for instance wish for their children to learn Arabic as well as Danish), as well as respondent driven sampling. The interlocutors included in this study thus vary in the way they think of and perform their identity as Muslims and in their affiliation to religious organizations.

The interviews focused on two overarching topics. The first topic explored indirect experiences with hate crime, how interlocutors dealt with this, and with whom they talked about it. It was usually in this part of the interview that narratives of direct experiences of hate perpetration where brought up as well. The second part of the interview focused more on interlocutors’ social connections and senses of belonging. The themes presented in the analysis below have been developed in a dialectic process between readings of the interview material and scholarly literature on, especially, epistemic injustice. Initial readings of the empirical material pointed toward a recurrent theme of how to interpret and communicate about hate incidents. Readings of the scholarly literature on epistemic injustice then focused our attention more specifically toward narratives relating to different epistemic practices regarding hate perpetration in the empirical material. The analytical themes and selected excerpts presented in this article, are thus a result of such a dialectic reading of the empirical material with a conceptually guided attention toward epistemic practices.

Finally, the choice of terminology (hate crime, hate incident, hate motivated assault) is not irrelevant. Our aim is to explore the feelings of (un)safety that emerges from knowing and sharing knowledge about *hate crime*. So this is where the conversation started. Initiating a conversation about hate crime has indeed elicited many important stories from our interlocutors. However, a crucial aspect of these stories is uncertainty: uncertainty about whether the experiences that come to mind are indeed examples of hate crime; uncertainty about what it takes for something to count as a hate crime; whether experiences are ‘serious enough,’ or whether the motivation behind the actions are of the relevant kind. So while hate crime – a term that immediately locates the conversation in a certain legal and criminological terrain – has been the point of departure, it is not on all occasions the most illuminating term for the actual experiences conveyed and the content of the knowledge explored below. In what follows, we restrict the use of the term ‘hate crime’ to the situations, where this word is used either in an interview question or answer. Otherwise, we apply terms such as ‘hate incident,’ ‘assault’ or ‘event’ in order to dislodge the conversation from any legal determination.

## 6 Hate crime in a Danish legal and political perspective

Policy and legislation regarding hate crime, racism and antisemitism has been on the political agenda in recent years in Denmark. Hate crime is legally covered under two sections of the penal code in Denmark, sections 86.1, a sentence enhancement statute, and 266b, a hate speech statute. In 2021 after intense public debate about the inadequacy of the existing legal framework section 81.6 was changed in order to lower the threshold for recognizing a bias motive (Retsinformation, 2021). Another way hate crime has proved to be on the political agenda in Denmark is through the political adoption of action plans. In January 2022, the Ministry of Justice released the “Action plan against anti-Semitism,” with 15 initiatives to prevent and combat antisemitism in Denmark. Among other things, the action plan initiatives cover education in elementary and high schools, security for Jewish institutions, more research on antisemitism, and focus on combating antisemitism through Danish foreign policy (Justitsministeriet, 2022). Shortly after the release of the action plan, the Parliament adopted a proposal to draw up a similar action plan against racism in Denmark. This action plan has yet to be released, but is, according to the Minister of Justice, expected to be released in 2024 (Folketinget, 2024).

One of the arguments for political action and legal change regarding hate crime in Denmark has been the discrepancies in measurements of the number of hate crimes. The Danish Crown Police publishes a yearly report on hate crime in Denmark. The latest report shows, that the Danish police registered 487 hate crimes in Denmark in 2021. Of these 300 were registered as racially motivated and 101 as religiously motivated, of which 50 targeted Muslims (Rigspolitiet, 2024).^2^ The number of hate crimes registered by the Crown Police is relatively low compared to the bi-annual victim survey conducted by the Ministry of Justice. In the latest survey from the Ministry of Justice, it is estimated that 20.000–31.000 persons in Denmark experienced being subjected to a hate crime between 2020 and 2021 across protected identity categories (Justitsministeriet, 2022). There can be several reasons for the discrepancy between the numbers of the victim survey and the cases registered by the Crown Police, including different perceptions of what counts as a crime, lack of trust in the police and legal system’s ability to handle hate crime (Atak, 2022) or a normalization of experiences of hate crime (Perry and Alvi, 2011). In any case, the discrepancy points toward a difficulty in making ends meet regarding experiences of hate crime and public recognition and sanctioning. It is within the context of this difficulty that the following stories about how to handle knowledge about hate crime and hate incidents should be interpreted.

## 7 Results: “maybe she was just angry?”

We begin this section with a narrative told by a woman, Zahra, in her late thirties. The narrative traces a course of events that are exemplary of the central themes to be explored below, and we therefore include the narrative in its entirety. It begins with an event, which Zahra in the moment interprets as a hate or bias motivated assault on the bus. Her immediate understanding of the situation is fairly certain: this is wrong and could be reported to the police. However, as the narrative proceeds, it becomes clear how her subsequent interactions (with a passenger on the bus, her colleagues and a police officer) calls her initial interpretation into question:

*I’ve tried, myself, to be [pauses] hit on the bus.*

*Uhm [pauses] it was on a bus from [not clear] to Frederiksberg. And it was very sudden, I got, like, an umbrella on the head. Then I went, I turned around, and it was a woman who had hit me. So I said, ‘was it you?’ ‘Yes’. Because she thought that the headscarf provoked her. Then she just started on a whole tirade that someone like me shouldn’t be here and should be ashamed to wear this. And it was like, you’re against the Danish society and women’s rights and all sorts of things. It was like she said a lot of things all at once. It seemed like she had a lot on her mind. And then she got off (…).*

*I was pretty shocked; I didn’t know what to do. And then I looked at another woman and said, ‘if I report it [to the police] will you be a witness to it?’, then she said, ‘no I won’t’, she didn’t want to take part in this nonsense, she said. So then, all of a sudden, I felt I was all alone in the bus. So that was pretty… (…).*

*But I also told my colleagues about it and just thought [pauses] it was a little awkward to say it because I had just started something new [a new job], right? And I also wanted to give a different impression of myself. And secondly, it’s also a little sensitive to talk about racism [not clear]. You have to be careful you’re not the one who talks about racism all the time. It’s a bit of a taboo to talk about at work. (…) It’s unprofessional. It’s like a bad thing you don’t talk about because when I said it, I felt that a lot of them became, like, a little uncomfortable. You know… (…) There are many who thinks, ‘it’s terrible, I think maybe you should see a therapist’, I didn’t think it was [not clear] I don’t know. I just felt a little, maybe I was just a bit too sensitive. (…).*

*But yeah, I reported it to the police, but there was someone, and well they said they would make a case of it, but I haven’t heard from them. I did want to have it reported so it would become part of the statistics too. I don’t know if it ever became a case. Hate crime, it is very [pauses] very hard to prove hate crime [said in English] or hate crime [said in Danish: hadforbrydelser] [not clear], but then at least I’ve tried. So I don’t know if it became [part of the] statistics. (…) No, I haven’t heard anything further. They said that they could maybe look at the [surveillance] recordings but they couldn’t see anything [not clear because of the wind] it was full, that is, the bus was full. So he [the policeman] said something about that there could be many other reasons. Maybe she wasn’t, you know, maybe she was just angry. An angry woman. (…).*

*But I think it’s not good enough [laughs]. To say something like that. But you know, it’s not really, he can’t really, well maybe she was, after all, an angry woman. You know, that’s really where, that’s why, there are many times where I doubt myself. And ‘maybe they are just angry or?’ I like give a little, you get a little like gaslighted or you… I don’t know if it’s gaslighting, but I just think, sometimes, there are many times where I doubt myself. Whether my feelings are right. Is it maybe just me who is overly sensitive?*

In the interaction with the woman on the bus, what emerges is simply a dismissal of Zahra’s interpretation (‘this nonsense’). Other interactions are more subtle, toned by hesitation, awkwardness (her colleagues) or suggestions that diminish the bias motive (‘maybe she was just an angry woman’ as suggested by the police). The reactions from colleagues and the police could be seen as attempts to qualify Zahra’s initial understanding. However, they could also – and, we would argue, more appropriately – be seen as trivializing her experience. The result seems to be that through the series of testimonial encounters Zahra’s confidence in her own ability to pass judgment on what has happened is impaired, leaving her in a hermeneutic lacuna, where she has difficulties making her initial experience and reaction convincingly intelligible to herself. The story also provides insights into one possible risk in epistemic practices around hate crime and hate incidents: that subsequent interactions may add feelings of self-doubt and self-critique to an initial harmful experience. In the following, we aim to elaborate this brief analysis, tracing different implications of such epistemic practices around hate crime and hate incidents for feelings of safety.

The first part of the analysis explores the work that our interlocutors do in order to build up and manage epistemic resources, for example by trying out different terminologies, seeking knowledge about hate crime and hate incidents, and trying to make sense of their experiences through engaging in conversations with others. The second part of the analysis examines in more detail the risks our interlocutors run when trying to make their experiences intelligible to relevant others in testimonial exchanges. The analysis unpacks how people navigate these risks by moderating their speech and weighing the potential gains and losses of speaking about their experiences.

### 7.1 Epistemic confidence and senses of hermeneutic safety

As Zahra’s narrative shows, making experiences of hate crime and hate incidents intelligible to oneself as well as others depends in part on having appropriate and collectively recognized hermeneutical resources available to talk about what has happened. However, such resources are never simply ‘in place.’ Whether or not a certain terminology is useful to make sense of one’s experiences depends not only on one’s own assessment of whether the word is fitting. It is also highly dependent on whether this assessment is recognized by relevant others, for example public authorities. One of the young women in this study reflected during the interview on whether it made sense for her to call an incident a hate crime, if it was not recognized as such by the police and the legal system:

*Sahar: I don’t know if it’s, like, just in my head, but I think maybe, it’s this way of thinking that probably nothing will be done about it until it becomes physical, right? That is, I think it is this way of thinking that means that in my head, then, hate crime is about when it becomes a physical, you know, a physical attack or something. I just can’t, well of course if I have experienced something racist on the bus, like I once did, then I wouldn’t call it a hate crime, even if it actually is a hate crime. There’s someone who is, like, standing and discriminating me in some way. But then I would probably not say ‘I have been subjected to a hate crime’. Then I would say, I have been subjected to something racist or something discriminating or something or other. I can see why it is called hate crime, but it’s just that it’s called ‘crime’ and nothing is done about that crime, it’s like. It sort of gets… Well, it’s actually kind of weird, right?*

*Interviewer: Yes, that’ll be weird.*

*Sahar: If someone has (…) [committed a] crime. But that you according to the law, you know like, that according to the law nothing is done about it, like, anything in practice, where you can say, ‘okay, this police report has gone through’ or this, ‘I have received an update on what happened with this person and this case’. Then I wouldn’t call it a hate crime. Then I would say that I have been subjected to some hate. I have been subjected to something discriminating, something racist. That is why I feel that way, I feel a bit weird about that word. Actually.*

(Sahar, woman, interview February 2022).

Obviously, some societal actors have superior authority in assessing the appropriate use of specific words in specific contexts. For example, the police and courts of justice have superior authority, when it comes to the appropriate use of the word ‘crime’ within the legal system. However, upon a close listening, what seems to be at stake for Sahar is not merely that she recognizes the authority of the police and courts to determine whether a hate crime has occurred. At stake is rather a pragmatic negotiation of meaning: it only makes sense for her to call it a crime, if the police and the courts perform the acts appropriate for a crime. She acknowledges why it is called ‘hate crime,’ however, if it is a *crime*, then why is nothing done about it? What is the point of insisting on calling it a hate crime, if it is not recognized as such by the police and legal system? Consequently, she opts for other terms that supposedly can grasp her experiences, such as discrimination, racism or hate. The uncertainty about whether the police takes hate motivated assaults seriously and the feelings of unsafety this may perpetuate is a recurring theme in the hate crime literature (for example Wickes et al., 2016; Atak, 2022). What is important for our investigation is the epistemic ramification of this uncertainty, namely that Sahar adjusts the terminology to fit the police’s work rather than staying with her own intuition (‘if someone has committed a crime’) or reproach the authorities for not doing enough. By aligning with the authorities, a certain fit is indeed created between word and world, which makes Sahar avoid the ‘weirdness.’ However, there seems to be a cost to this fit that lingers between the lines, namely a lack of appropriate recognition of a problem.

Gaining resources to recognize and speak about hate crime and hate incidents can also take a lot of emotional effort. This causes some people to work out different ways to take care of themselves emotionally while getting the information they need in order to stay informed. This is expressed in the excerpt below from the interview with Farah:

*Farah: Well, it’s not that I, like, consistently choose not to see them [online videos of hate crime], because I do. Also because this is the reality. You know, it’s how things are and I’m not normally the type to close my eyes to that sort of thing. Then rather be confronted with what the status quo is.*

*(…)*

*Interviewer: If you were to try and describe, what kinds of feelings or thoughts does it spark?*

*Farah: Mhm [pauses]. Mmmm. First [pauses] I think as a rule I try to form a general idea. What is it about? What has happened? And sometimes I succeed and other times you have to watch the video first. That is, then it doesn’t help to look at the commentary track or there is not that much to get from the captions, which have been written.*

*Interviewer: So sometimes, the solution is to read a bit around the video?*

*Farah: Yes, to just like try and see what it is. But then it’s maybe just people’s reactions and there isn’t that much of a description of the incident. And then I think I, like, consciously slash unconsciously make, like, an evaluation, is this something I feel up to right now? Is this something I want to deal with or not? If it’s not something I want to deal with. And there can be one of two reasons for that. It can be because either I don’t feel well, or it can be because I’m just in a mood or don’t feel like any negativity. But it can also be because I’m just so happy and I don’t want it to be ruined …*

(Farah, woman, interview February 2022)

What becomes evident here is how the benefits of knowing (‘not closing the eyes’, ‘this is reality’) constantly has to be balanced by a need for emotional self-care. Gaining hermeneutic resources comes with a price, which causes Farah to consider how to approach the videos, for example reading the commentaries first in order to be prepared, or choosing not to watch the videos at all in order to care for her own mood. During the interview, Farah also reflects on how new hermeneutic resources can make the world appear in a different way:

*Farah: Many years ago I was confronted with, like, the internalized racism I, like, had in my head. If I saw some young guys who just had too much energy but had brown skin and were noisy and then I thought, ‘that is just not good enough and they haven’t been raised properly at all and can’t they just, you know, can’t they make an effort, after all they’re in a public space now, or really we’re sitting in an S-train, it’s just so embarrassing.’ And one thing and another. Whereas if I saw the same group but just white guys, then I would think, ‘well, they’re just having fun, it’s a Friday night after all, they have had too much to drink or something, they’re just in a good mood’. So it wouldn’t be viewed as something negative in the same way. Still a source of irritation, but ‘they are just young, you know, they should be allowed to take up some space’. So, then I was confronted with that. And now I try to…*

*Interviewer: How was it?*

*Farah: It was because I had, or have some friends who, like, know a lot about, you know, one was studying Danish, the other studied psychology. And they were a lot, like, into these social topics and looked at it from a linguistic perspective and, like, psychological perspective. And they talked a lot about racism and at that time I couldn’t even talk about it because I just didn’t want to. It would be too all-encompassing and I’d rather just avoid it. But then, as I started to take more of an interest in it and I sort of started to acknowledge that it, like, existed and was confronted with it, well, then of course I could see it. So, but it also just means that you can’t go around and be, like, blissfully ignorant. All of a sudden, you just see the world differently… Yeah, so it was both good and bad [pauses].*

(Farah, woman, interview February 2022)

At the heart of this excerpt is a story about gaining new hermeneutic resources through supportive social relations. These resources help Farah to confront what she now sees as internalized racism, and which has hitherto caused her to misinterpret events in her surroundings. This part of the story could be seen as a situation in which hermeneutic safety is created: that Farah now can feel relatively secure in her ability to render her experiences communicatively intelligible through shared hermeneutical resources, and that people around her took an interest in helping to create them. However, the final part of the quote modifies this reading by reminding us that gaining such new insight about the world is not simply positive. While ignorance is typically considered problematic or even a vice epistemically speaking (see for example Medina, 2013), it can also, in some instances, seem ‘blissful’ – not least when the truth is burdensome. Farah is not alone in articulating that knowing can become ‘too all-encompassing’. Hence, the interlocutors often seek to weigh how much ‘space’ they want to give to the emotional and epistemic work implied in knowing about hate incidents in order to maintain space and energy for other aspects of life.

In the example above, the new knowledge came about through and was supported by particular social relations. An important part of having a sense of hermeneutic safety is this shared character of hermeneutic resources. What, then, does it entail to have or gain knowledge about the world that is different from conventional knowledge in the surrounding society? A consequence may be that it becomes difficult to convey one’s experiences and reactions to those who do not know the same things (a topic going back at least to the work of Goffmann and Tajfel on minoritized identities). Several of the interlocutors in this study reflect on how they navigate this epistemic difficulty by choosing hermeneutically safe relations, through which to share their experiences. This particularly applies to the younger interlocutors who are raised in Denmark, and who share a particular set of experiences as persons growing up as Muslims in a non-Muslim majority society. Amal describes this succinctly:

*Interviewer: Is there a difference in who you would tell about such an experience?*

*Amal: Yes. Well, in general I mostly have friends with a different ethnic background [than Danish]. So it’s a conversation there, you know, so I would probably tell it to them. I don’t know how much I would talk to my Danish friends about it. That is, then it would sound as if I’m standing there and play the victim role, really, in some way or other, right? So I don’t like to have conversations like that, because I like it to come naturally. That is, there is no difference between us. So I avoid talking about these topics in a way.*

*Interviewer: So with your friends who have…*

*Amal: A different background.*

*Interviewer: Yeah, then it’s easier? Or they understand more? Or?*

*Amal: Yes, they can relate to it of course. (…) If it’s ethnic Danes, then it’s [pauses] then they would say, ‘oh, that’s bad’ or, you know, ‘oh, that’s tough on you’, in some way or other, right? Because it’s not something they will go through. So with my ethnic friends, with another ethnic background, they would be more, like, think about themselves too. So it would be a completely different conversation you have in that way, right?*

*Interviewer: (…) Can you try to elaborate on these conversations you have with, like, or how they would play out with one and with the other? If that makes sense?*

*Amal: Yes, really, with one, that is with Danes, it might be that [pauses] the conversation will end quickly. Otherwise it would feel as if, well okay [pauses] I don’t really know how to explain it otherwise. But really, with my non-ethnic [minority ethnic] friends, then it will just be something they relate to, as I mentioned, and they will be able to understand even more because they themselves could have been subjected to it, and then you have the same situations that you have maybe been through and then you start to talk about that. But on the other hand, then it will be something that they [ethnic Danes] can’t relate to at all, because they are, you know, really from here, you could say, right?*

(Amal, woman, interview September 2023)

Such hermeneutically safe relations can serve two interlinked purposes. For one thing, sharing what Dotson calls risky testimony in such a relation is safer, because you are more likely to be believed. We will return to this in the second part of the analysis. Further, as the relevant others in such relations can relate to the experiences, it arguably becomes easier to make them comprehensible for oneself and others. This is especially important, when collective hermeneutical resources are not clearly established. Thus, sharing one’s experiences in hermeneutically safe relations can strengthen epistemic confidence, which again is likely to foster a development of hermeneutical resources. Thus, such practices can have the opposite effect of what Fricker describes as a felt “dissonance between received understandings and your own intimated sense of a given experience,” which “tends to undermine your faith in your own ability to make sense of the world, or at least the relevant region of the world.” (Fricker, 2007, p. 164). The flipside of this kind of safety is of course that people are pushed to share their experiences only within social contexts in which these experiences are confirmed, thereby avoiding important frictions in their epistemic encounters. This conundrum we will return to in the concluding remarks.

### 7.2 Testimonial risks – and what people do to handle them

In the initial narrative of this analysis, Zahra on several occasions tries to tell other people what has happened: to the other passenger, the colleagues, and to the police officer. In each of these situations, she – as anyone who engages in testimonial exchanges – confronts a risk; that she will not be heard as she hopes or expects to be heard. She expects to be heard as someone who truthfully recounts an experience, and not any experience, but an experience of something wrongful. However, in each linguistic encounter she experiences different forms of obstacles, from outright rejection to more subtle forms of questioning or doubt. We do not know the exact reasons behind each of these failed encounters, however they seem to display a systemic character that could indicate a credibility deficit that emerges through the combination of Zahra’s identity and the content of the testimony; that such a story told by ‘someone like her,’ is difficult to believe for certain people. Such a credibility deficit exacerbates the risk of failure in the linguistic exchanges, and several of the interlocutors are well aware of such risk (again pointing toward its systemic nature). One way of handling this risk is, as evidenced above, to choose one’s audience carefully when sharing risky testimony. Following Dotson, this careful selection of an audience could be seen as a response to testimonial quieting (Dotson, 2011). Another, and partly overlapping, way is through testimonial smothering. Here, a largely internal dialogue can take place, testing, so to speak, how much you think it is possible to tell others, more specifically, what you believe they are able or willing to hear. This process of “testing the waters” is expressed in the interview with Layal:

*Layal: (…) You test the waters. That is, where is this person? But I also think as someone… You know, it’s part of having a relation. Then you know a bit more in regards to where this person is at. Yeah, things like that. You know, it can prepare you. But in the end I think, do I want to have this conversation in the first place? Then you can get a chance to test the waters. Is it systematic? Because sometimes you can happen to say some things that are maybe not that clever. But I know that myself [laughs], like, I’m no exception. You just happen to say some things, where the first thing that comes to mind is said and maybe not worded very nicely. Yes, so it’s again that thing; test the waters. Yes. Then you find out bit by bit.*

(Layal, woman, interview September 2023)

As this excerpt shows, testimonial smothering functions as a way of navigating the risk and potential benefit of engaging in linguistic exchanges concerning risky testimony (Dotson, 2011). For the knower, it is a matter of discerning to whom one is speaking and what kind of testimony that person is able and willing to acknowledge. This may appear to be a sound or constructive way of navigating epistemic risk in a linguistic exchange. However, fundamentally it is a practice of silencing, as it emerges from the inability or unwillingness of the listener to hear the testimony (Dotson, 2011, p. 244).

Our interlocutors run an additional risk, when sharing experiences of hate incidents, which pertains to the way they are perceived by others. This is evident in the initial excerpt from Zahra, where she ponders whether she is over-sensitive. In another narrative about an incident at work, Zahra elaborates on this kind of risk:

*Zahra: (…) right before Corona I got a new job (…) I was really happy, of course. First day at a new job, new jacket and everything [laughs] like all, ‘yes’.*

*Interviewer: [Laughs] It was exciting.*

*Zahra: Exciting. And then I came in and she says, ‘yes, but the cleaning ladies, they are actually supposed to come in here’ [laughs] then I said, ‘oh no’. Then I said, ‘but I’m not a cleaning lady’. ‘Okay, aren’t you, I’ll just check, then I just need to call someone who will take you up’. ‘But I actually have an access code and the card to [the company]’. ‘Okay, yes, okay, of course I just had to make sure,’ right? You know, she would never have said that to my colleague if he were a white man, right? That he’s a cleaning lady [laughs]. So I told my new boss. And she’s American. And she’s also very aware of these things and said, ‘you know, you need to be careful you don’t become like the person with a chip on her shoulder’. Talks about racism all the time, right? That was the first thing she said to me [laughs].*

*Interviewer: That was what she said?*

*Zahra: ‘Be careful you don’t say it too many times, right?’*

*Interviewer: What do you think of that?*

*Zahra: Well, I think it was very unsympathetic, really. So I also told her that I hadn’t expected her to say [that], but I don’t have a chip on my shoulder, but I’m just saying that that was what happened. ‘But that’, she said, ‘is really very. Try and look around, who is it who cleans? Well, it’s people who look like you, you see that’s why’. But I said I do know that [laughs]. But that doesn’t, you know, doesn’t mean, then. I’m not mad at the receptionist, poor woman who just thought [not clear]. But you know, it was rather my boss’ reaction.*

(Zahra, woman, interview March 2022)

Besides confirming the importance of testimonial failure (what sticks for Zahra is not so much the initial incident as the inability of the boss to listen), the narrative presented here, describes the risk of being perceived as ‘the one who talks about racism all the time.’ Several of the interlocutors describe how they do a great deal of work to avoid such risk by ensuring ‘balance’ in their stories. This is partly in order to appear sober and therefore credible to listeners, not being the one ‘having a chip on my shoulder’ as Zahra describes it, or as someone who ‘plays the victim role’ as described by Amal. It is, however, also about creating a balance in their own everyday life and in how the world appears to themselves. The interlocutors do not want racism and hate incidents to taint everything. An example of how to create this balance is provided by Hiba, who very consciously and actively tries, together with her friend, to counterbalance the bad experiences and experiences of hate incidents with stories of good experiences and meetings:

*Interviewer: How, or what do you talk about when you talk about these topics?*

*Hiba: Yes, so I just want to remember the last thing we’ve talked about [pauses]. But once in a while it’s brought up that thing with, for example, should you wear a headscarf or should you not wear a headscarf? Or when they [in the media] talk about women who wear a headscarf or Muslims who wear a headscarf, that they are oppressed. And then we had a discussion, but had they asked everyone? How did she [Hiba’s friend] feel, how I felt about it when people talk like that. But luckily when you, for example the people I worked with. When people get to know you then it’s a whole other understanding of the topic. And it is [pauses]. So, but really I also think that we, you know, if I should say so myself, we’re pretty good at balancing. So when we talk about, ‘but that’s also just [makes a sound as someone grumbling about something:] urrgh-urrgh-urrgh-urrgh’, and then when we’ve calmed down, then we can also say, ‘buuut…’. So for instance at my old job I had a [name of female colleague] who was very sweet. ‘But she’s there too, and there is also this person, and there is also…’ And then all the good examples are brought up too and sort of make up for the things you experience once in a while.*

(Hiba, woman, interview May 2022)

Such acts of balancing can be understood as a form of emotional self-care, but also as a form of epistemic self-care: that the interlocutors protect their perception of their surroundings from eroding into overly negative or pessimistic assessments. Further, such balancing is not only conducted for the sake of the interlocutors themselves. In sharing stories of hate incidents with others ‘like themselves,’ several interlocutors express concern about the risk of passing on feelings of, for example, insecurity and vulnerability. Several interlocutors therefore talk about how they refrain from sharing stories and experiences of hate incidents with family members, friends and acquaintances, because they do not want to worry them. Bassam, who works at a school, tells about such epistemic care for others:

*Bassam: (…) But it’s another thing with kids [in his class] who come back and say ‘that was really’, you know, ‘she was pretty mean, that woman’ or, ‘also just so angry at us’, you know looked at them funny and that sort of thing. So, and that [pauses] yeah, that’s a shame.*

*Interviewer: How, like, do you talk with the kids about it then?*

*Bassam: Well, we do, yeah, we definitely do. You know, it’s of course typically the teachers who have been with them who takes [the conversation]. We have of course the policy to tell them that ‘there are some people who just have a harder time than others. There are maybe some people who have had some bad experiences. There are some people who are having a bad day.’ All sorts of things, like, trying to make it into a, well, an isolated situation. And a single person who maybe doesn’t exactly behave as they should. You can say that they [the kids] just have to not experience it too many times, because then it becomes difficult to believe in the story that it’s an isolated incident. We’ll just have to hope that they won’t, then. Because then they can begin to get the sense that there are just some people who don’t like us. And many of them, or more of them. And we try to avoid that as far as possible. It’s not something we can control in any way.*

(Bassam, man, interview March 2022)

As this quote shows, Bassam is conscious that he dismisses the students’ interpretation of events, even though he finds it accurate. However, he does this in the hope that it will help the students maintain a trusting or positive view of the world, if only for a little longer. There is a real attempt to care for the wellbeing of the other in this withholding of information, knowledge and experience, which the knower knows to be emotionally burdensome and epistemically distressing. The flipside of such care, however, is the impediment of hermeneutically safe relations and potentially the epistemic confidence of the students as described above. There is in way a troublesome mirroring between this way of managing the risks of sharing knowledge by making hate incidents into isolated events, and then the initial story told by Zahra which ends up with the conclusion that ‘maybe she was just an angry woman’. In order to protect others from knowledge that may change their perception of their surroundings and hence impair their sense of safety, the potential for developing collective hermeneutic resources for recognizing the systemic character of hate incidents is potentially undermined.

## 8 Conclusion: tightropes and trade-offs

In this article, we have investigated feelings of (un)safety emerging from knowing and sharing knowledge about hate crime or hate incidents among Muslims living in greater Copenhagen. We have combined research on the different types of harms of hate crime with insights from current philosophical reflections on epistemic encounters in order to highlight the distinct threats to senses of safety that emerge from epistemic practices around hate crime. We want to conclude this article by discussing how our findings contribute to each of these fields. Research on hate crime has investigated the psychological, emotional and behavioral consequences of hate crime, showing a range of implications for victims’ feelings of safety. Hate crime can, among other things, undermine victims’ self-esteem (Garnets et al., 1990), create diminished feelings of self-mastery and create feelings of heightened vulnerability to repeat victimization (Herek et al., 1999), and more generally foster feelings of unsafety, anxiety, and vulnerability, not least due to the intimidating message expressed in hate crime (Perry, 2001; Walters, 2022). Further, research suggest that the very fact of hearing or learning about hate crime against people with whom one identifies may perpetuate feelings of vulnerability (Noelle, 2002; Perry and Alvi, 2011; Walters et al., 2020). In this article, we build on this research but argue for the value of an added attention to the *epistemic* aspects of hate victimization. This is important, because of the distinctive risks one may encounter as a *knower*; that is as someone engaged in creating, formulating, and sharing knowledge. Paying specific attention to this aspect of victimization can provide us with a more nuanced picture of the consequences of hate crime, not just for victims (direct as well as indirect), but for a broader societal context.

Firstly, and confirming the existing literature, the very fact of knowing that ‘this can happen to someone like me makes the world feel less safe for our interlocutors. However, further risks then potentially emerge in and through subsequent epistemic encounters. When our interlocutors try to share knowledge about their own experiences of, for example, hate-motivated assaults or incidents, having that knowledge rejected or questioned create additional harms that relate more specifically to the nature of epistemic exchanges. In such exchanges, credibility and trustworthiness matter, and being rejected or doubted in such exchanges may create self-doubt and impairment of one’s confidence in passing adequate epistemic judgments. Conversely, when people are able to engage in successful epistemic exchanges, the ability to make one’s experiences intelligible can be strengthened. Thus, subsequent experiences with sharing knowledge – be it with authorities, colleagues, or witnesses – importantly shape the form and magnitude of harm.

The specific attention to the epistemic aspects of hate victimization may also expand our understanding of the way people sometimes alter their behavior in response to hate crimes. While existing research on the harms of hate crime has emphasized for example spatial and visual/aesthetic behavioral changes, such as avoiding certain places at certain times or hiding visual markers of a targeted identity (for example McDevitt et al., 2001; Perry and Alvi, 2011), different and distinct behavioral changes seems to attach to the handling of epistemic risks. Behavioral modification can here imply: choosing when to expose oneself to stories about hate crime, attempting to balance the burdensome knowledge with other soothing stories and experiences, or withholding knowledge from other people, either in order to protect oneself from dismissive responses or in order to protect the other person from the harm of knowing. Especially the latter practices have specific societal ramifications beyond the individual person who is altering their behavior.

When knowledge is selectively shared, it propels a risk of damage being done to the collective pool of knowledge about hate crime and hate incidents. Such damage to shared epistemic resources can occur, either because the development of adequate hermeneutic resources is hampered, or because epistemic ‘pockets’ are created where knowledge is only shared amongst people who already can identify with the experiences. However, if our interlocutors should work actively to counter this risk, they not only face the risk of rejection, but an additional risk of being seen as overly sensitive or overly occupied with racism etc., which again may damage their credibility. Such awareness of the potential threats to standing and credibility is important for a more profound understanding of the sense of social vulnerability that hate crime may perpetuate.

Moving in this terrain seems indeed to imply walking a tightrope between different risks, and not surprisingly, then, our investigation points toward a continuous work of *balancing* undertaken by our interlocutors. Such balancing is external (adjusting one’s conversations with others, assessing how much they are able to hear, and managing how one appears to others) as well as internal (balancing one’s own occupation with the topic, for example by engaging positive ‘counter-evidence’ in order not to become ‘swallowed up’ by it). It is tempting to emphasize the value in sharing important and difficult knowledge for example about hate incidents or other forms of wrong that seem to emerge along structural lines. Such emphasis is also found in the second field of literature on which we draw in this article, which is largely dedicated to highlight the risks of epistemic vices such as ignorance and narrowmindedness (Medina, 2013, p. 30ff) and epistemic injustices such as not being adequately heard (Fricker, 2007; Dotson, 2011). The ability to recognize wrongs, testify about them, and built up publicly available knowledge is of course crucial, and surely, there are both courageous and liberating moments in ‘speaking truth to power.’ However, entering the conversation through a sense of safety lens brings forth some of the costs of doing so. When qualitatively unpacking the harms and risks at stake in epistemic practices about hate crime and hate incidents, what emerges are difficult trade-offs between different needs. There is the needs to know accurately and to share that knowledge with others, and then there is the need to protect one’s emotional well-being, one’s loved ones, and the conviction that society is largely fair and good (the “adaptive illusion of personal invulnerability and relative safety” mentioned by Herek and colleagues). This is not an easy task, and knowing and navigating knowledge about hate crime and hate incidents from a minoritized position is indeed in itself a risky endeavor.

## Data availability statement

The dataset presented in this article is not readily available because data is not shared due to ethical and privacy restrictions. Requests to access the datasets should be directed to bjohansen@hum.ku.dk.

## Ethics statement

The studies involving humans were approved by the Research Ethics Committee at the Faculty of Humanities, University of Copenhagen. The studies were conducted in accordance with the local legislation and institutional requirements. The participants provided their written informed consent to participate in this study.

## Author contributions

AF: Conceptualization, Data curation, Formal analysis, Investigation, Methodology, Writing – original draft, Writing – review & editing. BSJ: Conceptualization, Formal analysis, Funding acquisition, Methodology, Project administration, Writing – original draft, Writing – review & editing.

## Footnotes

^1^Obviously, some persons may feel safe in any testimonial encounter, because they are overly confident that they are right, or because they are used to being seen as superior knowers (see Medina, 2013 for an elaboration of such vices). Feeling safe in this way may be ethically problematic, not least because it is likely to create unsafety in linguistic exchanges for other people.

^2^Researchers estimate that there were app. 300.000 Muslims in Denmark by the end of the 2010’s, corresponding to app. 5 percent of the Danish population (Kühle and Larsen, 2019, p. 68).
